# Phenotype-specific therapeutic efficacy of ilofotase alfa in patients with sepsis-associated acute kidney injury

**DOI:** 10.1186/s13054-024-04837-y

**Published:** 2024-02-19

**Authors:** Niklas Bruse, Katerina Pardali, Maarten Kraan, Matthijs Kox, Peter Pickkers, the REVIVAL investigators

**Affiliations:** 1 Department of Intensive Care Medicine, Radboud University Medical Center, Nijmegen, The Netherlands; 2 AM-Pharma B.V., Utrecht, The Netherlands

**Keywords:** Sepsis, Acute kidney injury, Chronic kidney disease, MAKE90, Phenotypes, Machine learning, Cluster analysis

## Abstract

**Background:**

There is no effective treatment for sepsis-associated acute kidney injury (SA-AKI). Ilofotase alfa (human recombinant alkaline phosphatase) has been shown to exert reno-protective properties, although it remains unclear which patients might be most likely to benefit. We aimed to identify a clinical phenotype associated with ilofotase alfa's therapeutic efficacy.

**Methods:**

Data from 570 out of 650 patients enrolled in the REVIVAL trial were used in a stepwise machine learning approach. First, clinical variables with increasing or decreasing risk ratios for ilofotase alfa treatment across quartiles for the main secondary endpoint, Major Adverse Kidney Events up to day 90 (MAKE90), were selected. Second, linear regression analysis was used to determine the therapeutic effect size. Finally, the top-15 variables were used in different clustering analyses with consensus assessment.

**Results:**

The optimal clustering model comprised two phenotypes. Phenotype 1 displayed relatively lower disease severity scores, and less pronounced renal and pulmonary dysfunction. Phenotype 2 exhibited higher severity scores and creatinine, with lower eGFR and bicarbonate levels. Compared with placebo treatment, ilofotase alfa significantly reduced MAKE90 events for phenotype 2 patients (54% vs. 68%, *p* = 0.013), but not for phenotype 1 patients (49% vs. 46%, *p* = 0.54).

**Conclusion:**

We identified a clinical phenotype comprising severely ill patients with underlying kidney disease who benefitted most from ilofotase alfa treatment. This yields insight into the therapeutic potential of this novel treatment in more homogeneous patient groups and could guide patient selection in future trials, showing promise for personalized medicine in SA-AKI and other complex conditions.

**Supplementary Information:**

The online version contains supplementary material available at 10.1186/s13054-024-04837-y.

## Introduction

Sepsis-associated acute kidney injury (SA-AKI) is a challenging condition with serious short- and long-term consequences [1] and lack of effective treatments [2]. The phase 2 ‘STOP-AKI’ trial evaluated the potential of ilofotase alfa (human recombinant alkaline phosphatase) in 301 SA-AKI patients, showing long-term renal function improvements and significantly reduced mortality [3]. Subsequently, the phase 3 global ‘REVIVAL’ trial, with 28-day all-cause mortality as the primary endpoint, was discontinued early due to futility [4, 5]. However, ilofotase alfa did show therapeutic efficacy on the main secondary endpoint, Major Adverse Kidney Event up to day 90 (MAKE90) [4, 5]. This is consistent with findings obtained in two earlier studies using bovine alkaline phosphatase [6, 7], and with the STOP-AKI study results [3]. In addition, patients enrolled in REVIVAL who had pre-existent renal impairment, e.g., chronic kidney disease (CKD), showed more therapeutic efficacy of ilofotase alfa, illustrating the potential of a personalized treatment approach [5]. Clinical phenotyping is a machine learning-based approach which has been shown to identify more homogeneous subgroups within diverse patient populations [8, 9]. As such, it may resolve patient heterogeneity and provide opportunities for personalized treatment approaches.

In the present study, we aimed to identify a clinical phenotype associated with ilofotase alfa's therapeutic efficacy in patients enrolled in the REVIVAL trial. This approach yields insight into the therapeutic potential of this novel treatment in more homogeneous patient groups and may aid more precise patient selection for future trials.

## Materials and methods

### Patients

We performed a post-hoc analysis on patients enrolled in the phase 3 REVIVAL trial studying the effects of ilofotase alfa in patients with SA-AKI. In total, 650 patients were enrolled into the trial of whom 329 received 1.6 mg/kg ilofotase alfa and 319 received placebo. The study protocol, detailing all in- and exclusion criteria and procedures, as well as the overall results of the trial were previously published [4, 5]. For the current analysis, patients with confirmed COVID-19 (*n* = 33), or those who had received renal replacement therapy prior to study drug administration (*n* = 23) were excluded, leaving 592 patients for further analyses. The ensuing workflow is graphically depicted in Additional file 2: Fig. S1 and detailed below.

### Variable selection

MAKE90 was defined as mortality through day 90, or an estimated glomerular filtration rate (eGFR) drop of > 25% at day 90 compared to pre-AKI value, or any RRT events through day 28 or RRT status at day 90. Forty-four numeric variables measured at study inclusion (immediately before study drug administration) were used as input for the variable selection algorithm. To select predicting variables for the therapeutic efficacy of ilofotase alfa, the baseline measurements of all variables were first categorized into quartiles. For each quartile a risk ratio was calculated using the formula:

$${\mathrm{Risk\, Ratio}}= \frac{{\text{CI}}_{\rm e}}{{\text{CI}}_{\rm u}}$$

Here, Cl_e_ is the cumulative incidence of the exposed group (ilofotase alfa) and Cl_u_ the cumulative incidence of the unexposed group (placebo). Consequently, the selected parameters are enriched for a potential relationship with the therapeutic efficacy of the compound (although chance variation may also play a role), thereby optimizing the feature selection. If the risk ratios for a variable demonstrated a consistent increase or decrease (indicating a beneficial effect) in at least three consecutive quartiles, this implied a degree of therapeutic efficacy, and therefore the variable advanced to the next step. This selection process yielded 32 variables (Additional file 3: Table S1). Next, we gaged the effect size of the effect of the selected variables by calculating the slope of the sequential increase or decrease in risk ratio using linear regression models. For variables that showed a consistent increase or decrease in all four consecutive quartiles, we calculated two separate slopes (one from the first to third quartile and another from the second to fourth). Subsequently, slopes were ranked by steepness, with steeper slopes implying more pronounced therapeutic efficacy, and the 15 highest ranked variables were used for the subsequent clustering analysis (see next section).

### Model selection

Models for all possible variable combinations were constructed using consensus clustering (ConsensusClusteringPlus package) for k-means and Partition Around Medoids (PAM) [10]. Patients with more than 50% missing data in one or more of the variables used in each model were excluded prior to clustering. For each model, missing data were imputed using the missRanger package, which employs a chained random forest approach [11, 12], and log-transformation, scaling, and centering were applied. Finally, the Bonferroni Outlier Test was used to identify and remove outlier patients in each model.

To assess each model’s robustness and quality, we employed the package’s cluster-consensus score [13]. Only models with a score > 0.90 for both phenotypes were retained. In retained models, differences in the incidence of MAKE90 events between the ilofotase alfa and placebo group over time were determined for each phenotype using log-rank tests. Models were included in the final selection if at least one phenotype showed a significant difference in MAKE90 events. From this list, the model that illustrated the strongest separation in the phenotype displaying a significant benefit for the patient group that had received treatment with ilofotase alfa was chosen for further analysis.

### Statistical analysis

Data are presented as median [interquartile range] or number (%). Differences in patient characteristics and outcomes between the phenotypes or between ilofotase alfa- and placebo-treated patients were tested using Mann–Whitney *U* tests and Chi-squared tests. All analyses were performed in R 3.6.3.

## Results

After removal of 22 patients with outlier values, 570 patients remained available for analysis, of which 358 (63%) were male, age was 70 [62–76] years, BMI was 28.2 [24.7–33.3], and APACHE II score was 23 [18–28] (Additional file 4: Table S2).

**Fig. 1:**
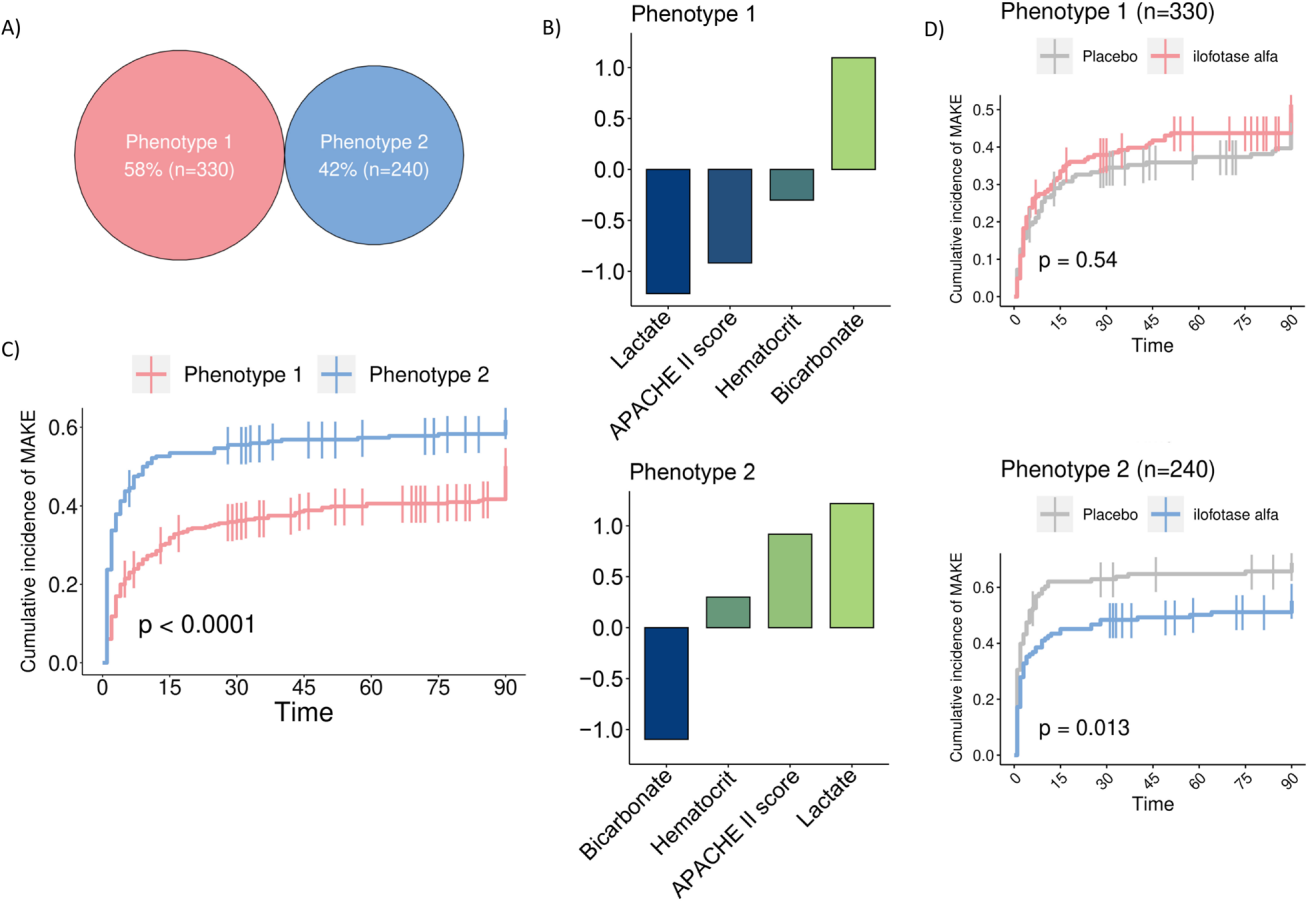
Phenotype characteristics and outcomes. **A** Distribution of the two identified phenotypes, **B** Standardized mean difference (SMD) of all clustering variables per phenotype, illustrating how far each variable for that group is removed from the mean of the entire cohort, **C** Cumulative 90-day incidence of MAKE events for the two phenotypes. **D** Cumulative 90-day incidence of MAKE events for ilofotase alfa- and placebo-treated patients within the two phenotypes. *p* values were calculated by log-rank tests

The model yielding the optimal fit consisted of k-means clustering with 2 phenotypes, including the variables *APACHE II score, bicarbonate, hematocrit,* and *lactate* (Table 1, Additional file 5: Fig. S2). Phenotype 1 (*n* = 330, 58%) comprised patients who were less severely ill, indicated by significantly lower APACHE II and mSOFA disease severity scores and lactate, as well as higher eGFR and PaO_2_/FiO_2_ ratios (Table 1, Additional file 4: Table S2, Fig. 1A, B). Reciprocally, phenotype 2 (*n* = 240, 42%) included patients with more severe disease and corresponding laboratory derangements. It should be noted that these phenotypes are based on variables present at inclusion, and therefore on pre-existing kidney function. There were no differences in CKD (assessed by pre-AKI eGFR) between the two phenotypes (Additional file 4: Table S2).

**Table 1:** Patient characteristics and outcomes of the different phenotypes and treatment groups

	Phenotype 1			Phenotype 2		
	Ilofotase alfa (*n* = 164)	Placebo (*n* = 166)	*p* value	Ilofotase alfa (*n* = 122)	Placebo (*n* = 118)	*p* value
Age (years)	71.00 [64.00, 76.00]	70.00 [61.00, 76.75]		69.00 [61.25, 76.00]	69.50 [60.00, 77.00]	
BMI	29.19 [24.41, 33.46]	27.26 [25.13, 34.92]		28.55 [25.38, 32.69]	26.96 [24.57, 32.54]	
Sex (% males)	105 (64.0%)	110 (66.3%)		73 (59.8%)	70 (59.3%)	
Pre-AKI eGFR (mL/min/1.73 m2)	74.08 [59.77, 90.22]	74.98 [64.29, 89.96]		74.87 [61.59, 87.22]	72.13 [53.99, 86.86]	
APACHE II score	19.50 [16.00, 25.00]	21.00 [17.00, 24.25]		27.00 [22.00, 32.00]	26.00 [22.00, 30.75]	
mSOFA score	8.00 [7.00, 10.00]	8.00 [7.00, 10.00]		9.00 [8.00, 11.00]	10.00 [9.00, 12.00]	**
Bicarbonate (mmol/L)	22.00 [19.62, 24.60]	22.00 [20.00, 24.90]		17.05 [15.00, 19.00]	18.00 [16.00, 20.00]	
Hematocrit (%)	32.60 [28.70, 37.00]	33.00 [27.08, 38.15]		35.95 [30.00, 40.62]	34.30 [29.55, 39.05]	
Lactate (mmol/L)	1.70 [1.10, 2.15]	1.70 [1.20, 2.48]		3.90 [2.60, 5.40]	4.25 [3.00, 6.62]	
MAKE90 (%)	81 (49.4%)	76 (45.8%)		66 (54.1%)	80 (67.8%)	*
> 25% drop in eGFR at day 90 compared with pre-AKI reference-eGFR	12 (7.3%)	17 (10.2%)		7 ( 5.7%)	8 ( 6.8%)	
On RRT at day 90 OR on RRT through day28 (%)	35 (21.3%)	38 (22.9%)		42 (34.4%)	57 (48.3%)	*
28-day in-hospital mortality (%)	39 (23.8%)	31 (18.7%)		39 (32.0%)	42 (35.6%)	
90-day in-hospital mortality (%)	51 (31.1%)	43 (25.9%)		46 (37.7%)	51 (43.2%)	

Clinical parameters were measured right before administration of treatment. Underlined parameters were used for clustering. Data are presented as median [interquartile range], or number (%). * indicates *p* = 0.01–0.05, ** indicates *p* = 0.001–0.01, *** indicates *p* = 0–0.001 calculated by Mann–Whitney *U* tests or Chi-square tests across both phenotypes

APACHE II: Acute Physiology and Chronic Health Evaluation II, BMI: Body Mass Index, mSOFA: Modified Sequential Organ Failure Assessment, Pre-AKI: Measurements taken prior to the onset of AKI. AKI Diagnosis: Measurements taken at the timepoint nearest to the AKI diagnosis, Baseline: Measurements taken at the timepoint closest to study inclusion

Overall, patients with phenotype 1 demonstrated a statistically significantly lower chance for a MAKE90 event than those with phenotype 2 (21% and 34%, respectively, log-rank *p* < 0.001, Fig. 1C).

For both phenotypes, the proportion of patients who received treatment with ilofotase alfa and those who received placebo was virtually equal, and their characteristics were comparable (Table 1). Patients exhibiting the more critically ill phenotype 2 showed a notable benefit from ilofotase alfa treatment, with a MAKE90 event incidence of 68% compared to 54% in the placebo group (log-rank *p* = 0.01, Fig. 1D). This advantage was predominantly driven by the receipt of RRT (34% vs 48% for the placebo group, *p* = 0.04, Table 1). Conversely, patients with phenotype 1 did not show a benefit from ilofotase alfa treatment (MAKE90: 49% vs. 46%, log-rank *p* = 0.54).

## Discussion

In this study, distinct clinical phenotypes related to a renal endpoint and the therapeutic efficacy of a novel drug were identified. Patients with phenotype 2, displaying more severe disease and corresponding metabolic and respiratory impairment, were at the highest risk for a MAKE90 event and displayed a clear benefit from treatment with ilofotase alfa. For this phenotype, ilofotase alfa treatment was associated with a 14% decrease in MAKE90 events, an effect size greater than the 8% decrease previously reported in the undifferentiated patient population [5].

These results emphasize the potential value of clinical phenotyping on baseline characteristics in increasing the chances to detect a therapeutic effect. Such an approach may facilitate personalized medicine, particularly for complex conditions like sepsis and AKI. Directly related to the present study, our findings can inform the design of future trials investigating ilofotase alfa in patients with SA-AKI.

Three limitations deserve attention. First, due to the strict inclusion- and exclusion criteria, clinical trials such as the REVIVAL study include less variation than is observed in normal clinical practice. Therefore, phenotyping may lead to more pronounced differentiation when used in routine healthcare. Second, implementing our findings to patient selection for trials requires a shift away from traditional in- and exclusion criteria. The artificial intelligence and machine learning methods employed pose challenges in terms of creating understandable and usable models for healthcare staff. Lastly, being an exploratory study, further external validation would be required to ensure robustness of the identified phenotypes. Unfortunately, this was currently not feasible, as ilofotase alfa is a new compound that has thus far been studied in only a single phase 3 trial. The dose-finding phase 2 STOP-AKI study population has several limitations, such as an even smaller group of patients treated with the therapeutic dose of ilofotase alfa and high missingness in clustering variables [3].

In conclusion, our study underscores the potential of data-driven patient phenotyping in complex conditions such as SA-AKI. Identification of patient subgroups could facilitate the design of targeted and therefore more efficient clinical trials, inform therapeutic decision-making, and ultimately foster improved patient outcomes.

### Supplementary Information

**Additional file 1.** List of the REVIVAL investigators (Steering committee).

**Additional file 2: Fig. S1.** Overview of the algorithm. An overview of the work process of the used algorithm to identify the phenotypes.

**Additional file 3. Table S1.** Overview of the 32 variables selected for their therapeutic efficacy, ranked by the steepness of their slope.

**Additional file 4. Table S2.** Patient characteristics and outcomes of the different phenotypes and treatment groups, including all variables.

**Additional file 5: Fig. S2.** Consensus clustering using k-means. A) Relative change in the area under the cumulative distribution function (CDF) curve with increasing clusters (k), with little change beyond k = 2, B) Tracking plot across k = 2–6, C) Consensus CDF plot across.

**Additional file 6.** List of the REVIVAL investigators.

## Data Availability

The datasets used and analyzed in the present study are proprietary to AM Pharma B.V. Due to confidentiality agreements and proprietary restrictions, these data cannot be made publicly available.

## Abbreviations

SA-AKI: Sepsis-associated acute kidney injury
MAKE90: Major adverse kidney events up to day 90
CKD: Chronic kidney disease
eGFR: Estimated glomerular filtration rate
RRT: Renal replacement therapy
AKI: Acute kidney injury
APACHE II: Acute physiology and chronic health evaluation II
BMI: Body mass index
PAM: Partition around medoids
