# Supplementary material for: Phenotype-specific therapeutic efficacy of ilofotase alfa in patients with sepsis-associated acute kidney injury
Source: Crit Care. 2024 Feb 19;28:50. doi: 10.1186/s13054-024-04837-y (PMC10875769; doi:10.1186/s13054-024-04837-y)
Supplement: Supplementary file 1 — Additional file 1. List of the REVIVAL investigators (Steering committee). [file 13054_2024_4837_MOESM1_ESM.docx]

**Steering committee**

**Chair:**

Peter Pickkers, Department of Intensive Care, Radboudumc Nijmegen, The Netherlands

**Members:**

Derek C. Angus, Department of Critical Care Medicine, University of Pittsburgh School of Medicine, Pittsburgh, PA, USA

Kristie Bass, AM-Pharma, Utrecht, The Netherlands

Rinaldo Bellomo, Department of Intensive Care, Austin Hospital, Melbourne, VIC, Australia;

Australian and New Zealand Intensive Care Research Centre, Monash University, Melbourne, Australia

Erik van den Berg, AM-Pharma, Utrecht, The Netherlands

Juliane Bernholz, AM-Pharma, Utrecht, The Netherlands

Morten H. Bestle, Department of Anesthesiology and intensive Care, Copenhagen University Hospital – North Zealand, Hilleroed, Denmark; Department of Clinical Medicine, University of Copenhagen, Copenhagen, Denmark

Kent Doi, Department of Emergency and Critical Care Medicine, The University of Tokyo, Tokyo, Japan

Chistopher J. Doig, Department of Critical Care Medicine, Medicine and Community Health Sciences, Cumming School of Medicine, University of Calgary

Ricard Ferrer, Department of Intensive Care Medicine, Val d'Hebron University Hospital, SODIR-VHIR research group, Barcelona, Spain

Bruno Francois, Intensive care, Inserm CIC 1435 & UMR 1092, CHU Limoges, Limoges, France

Henrik Gammelager, Department of Intensive Care Medicine, Aarhus University Hospital, Aarhus, Denmark

Ulf Goettrup Pedersen, Intensive Care Unit, Zealand University Hospital, Koege, Denmark

Eric Hoste, Dept of Internal Medicine and Pediatrics, Intensive Care Unit, Ghent University Hospital, Ghent University, Ghent, Belgium, and Research Foundation-Flanders, (FWO), Brussels, Belgium

Susanne Iversen, Department of Anaesthesiology and Intensive Care, Slagelse Hospital, Slagelse, Denmark

Michael Joannidis, Division of Intensive Care and Emergency Medicine; Department of Internal Medicine, Medical University of Innsbruck, Innsbruck, Austria

John A. Kellum, Center for Critical Care Nephrology, Department of Critical Care Medicine, University of Pittsburgh

Kathleen Liu, Division of Nephrology, Department of Medicine, University of California, San Francisco, San Francisco, California

Melanie Meersch, Department of Anesthesiology, Intensive Care and Pain Medicine, University Hospital Münster, Münster, Germany

Ravindra Mehta, Department of Medicine, University of California San Diego, La Jolla, CA

Scott Millington, University of Ottawa/The Ottawa Hospital, Ottawa, Canada

Patrick T. Murray, School of Medicine, University College Dublin, Dublin, Ireland

Alistair Nichol, University College Dublin-Clinical Research Centre at St Vincents university Hospital, Dublin, Ireland; and Australian and New Zealand Intensive Care Research Centre, Monash University, Melbourne, Australia

Marlies Ostermann, Department of Critical Care, Guys & St Thomas' Foundation Trust, London, United Kingdom

Ville Pettilä, Department of Perioperative and Intensive Care, University of Helsinki and Helsinki University Hospital, HUS, Finland

Christoffer Solling, Department of Anaestesiology and Intensive Care, Viborg Regional Hospital, Denmark

Matthias Winkel, AM-Pharma, Utrecht, The Netherlands

Paul J Young, 1Intensive Care Unit, Wellington Hospital, Wellington, New Zealand; 2Medical Research Institute of New Zealand, Wellington, New Zealand; 3Australian and New Zealand Intensive Care Research Centre, Monash University, Melbourne, Victoria, Australia; 4Department of Critical Care, University of Melbourne, Melbourne, Victoria, Australia.

Alexander Zarbock, Department of Anesthesiology, Intensive Care and Pain Medicine, University Hospital Münster, Münster, Germany.

**REVIVAL-investigators:**

See attached Excell-file.

**Acknowledgments**

PI’s of all enrolling sites and part of the REVIVAL investigators are depicted in supplemental file 1. We thank all participating patients, their families for their support, as well as study coordinators and research nurses of all participating sites in delivering the REVIVAL trial.

**Funding**

This work was supported by AM-Pharma. The role of the sponsor in the design of the study was to coordinate and facilitate processes, where the scientific input was provided by the members of the steering committee, and specific input by external experts in data management and statistics. Data were interpreted by all coauthors. All coauthors reviewed, made adjustments, and approved the manuscript. The decision to submit the manuscript was made by the principal investigator and other coauthors.

**Conflict of interest statements:**

Peter Pickkers has received travel reimbursements and consulting fees from AM-Pharma in relation to his role as PI for REVIVAL, and consulting fees from Adrenomed, EBI Paion, Sphingotec, and 4Teen4 outside the submitted work.

Derek C. Angus has received consulting fees from AM-Pharma.

Kristie Bass, AM-Pharma BV, The Netherlands.

Rinaldo Bellomo has received consulting fees and research support from AM-Pharma, Baxter, Paion, Viatris, Jafron Biomedical, and CSL Behring.

Erik van den Berg, AM-Pharma BV, The Netherlands.

Juliane Bernholz, AM-Pharma BV, The Netherlands.

Morten H. Bestle has received consulting fees from AM-Pharma in relation to his role for REVIVAL and has conducted contract research for Inotrem outside of the submitted work.

Kent Doi has received consulting fees from AM-Pharma.

Chistopher J. Doig reports no conflicts of interest.

Ricard Ferrer has received consulting fees from AM-Pharma.

Bruno Francois has received consulting fees from AM-Pharma as a member of the REVIVAL steering committee, and consulting fees from Inotrem, Aridis and Enlivex outside the submitted work.

Henrik Gammelager reports funding from various companies in the form of research grants to (and administered by) Aarhus University or Aarhus University Hospital. H.G. has received support for attending meetings by Baxter A/S.

Ulf Goettrup-Pedersen reports no conflicts of interest.

Eric Hoste has received a travel grant from AM-Pharma.

Susanne Iversen reports no conflicts of interest.

Michael Joannidis has received honoraria or research support from Baxter Healthcare Corp, AM-Pharma, CLS Behring, Fresenius, Takeda, Sanofi and Novartis.

John A. Kellum discloses fees paid by AM-Pharma in relation to his role as national PI for REVIVAL and is currently a full-time employee of Spectral Medical.

Kathleen Liu has been a member of the REVIVAL Steering Committee for AM Pharma. She has been a consultant/member of the DSMB for Seastar, Novartis, BOA Medical, Baxter, and Biomerieux, and she holds stock in Amgen.

Melanie Meersch has received lecture fees from Baxter and Fresenius Medical Care.

Ravindra Mehta reports honoraria for consulting from Baxter, Biomerieux, Mallinckrodt, GE Healthcare, Sanofi, Abiomed, NovaBiomed, Renasym and advisory board reimbursements from AM Pharma, Renibus, Alexion, Novartis and Guard.

Scott Millington reports no conflicts of interest.

Patrick T. Murray has received consulting fees from AM-Pharma (for Clinical Trial Steering Committee activities), Novartis, Renibus Therapeutics, and Alexion.

Alistair Nichol reports an unrestricted grant from Baxter to support the renal sub study of the TAME trial.

Marlies Ostermann has received speaker honoraria from Fresenius Medical, Baxter and Biomerieux; her institution received research funding from Baxter, Fresenius Medical, Biomerieux and LaJolla Pharma.

Christoffer Solling reports no conflicts of interest.

Pettila Ville reports no conflicts of interest.

Matthias Winkel, AM-Pharma BV, The Netherlands.

Paul J Young has received consulting fees from AM Pharma and from Baxter Healthcare Pty.

Alexander Zarbock has received consulting fees from Astute-Biomerieux, Baxter, Bayer, Novartis, Guard Therapeutics, AM Pharma, Paion, Renibus, Fresenius, research funding from Astute-Biomerieux, Fresenius, Baxter, and speakers fees from Astute-Biomerieux, Fresenius, Baxter.
