# Supplementary figures and images for: Phenotype-specific therapeutic efficacy of ilofotase alfa in patients with sepsis-associated acute kidney injury
Source: Crit Care. 2024 Feb 19;28:50. doi: 10.1186/s13054-024-04837-y (PMC10875769; doi:10.1186/s13054-024-04837-y)

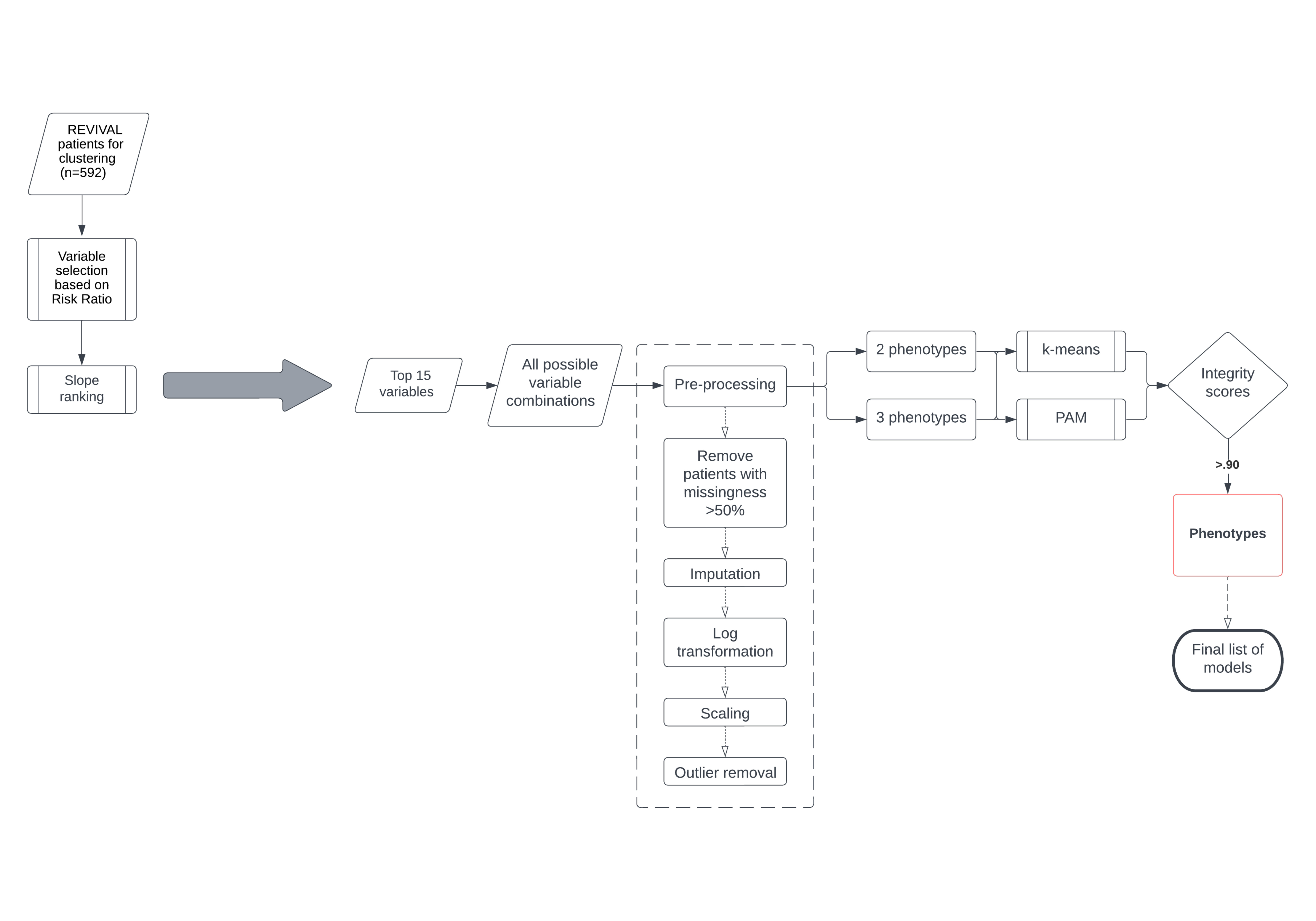

Supplement: Supplementary file 2 — Additional file 2: Fig. S1. Overview of the algorithm. An overview of the work process of the used algorithm to identify the phenotypes. [file 13054_2024_4837_MOESM2_ESM.jpg]

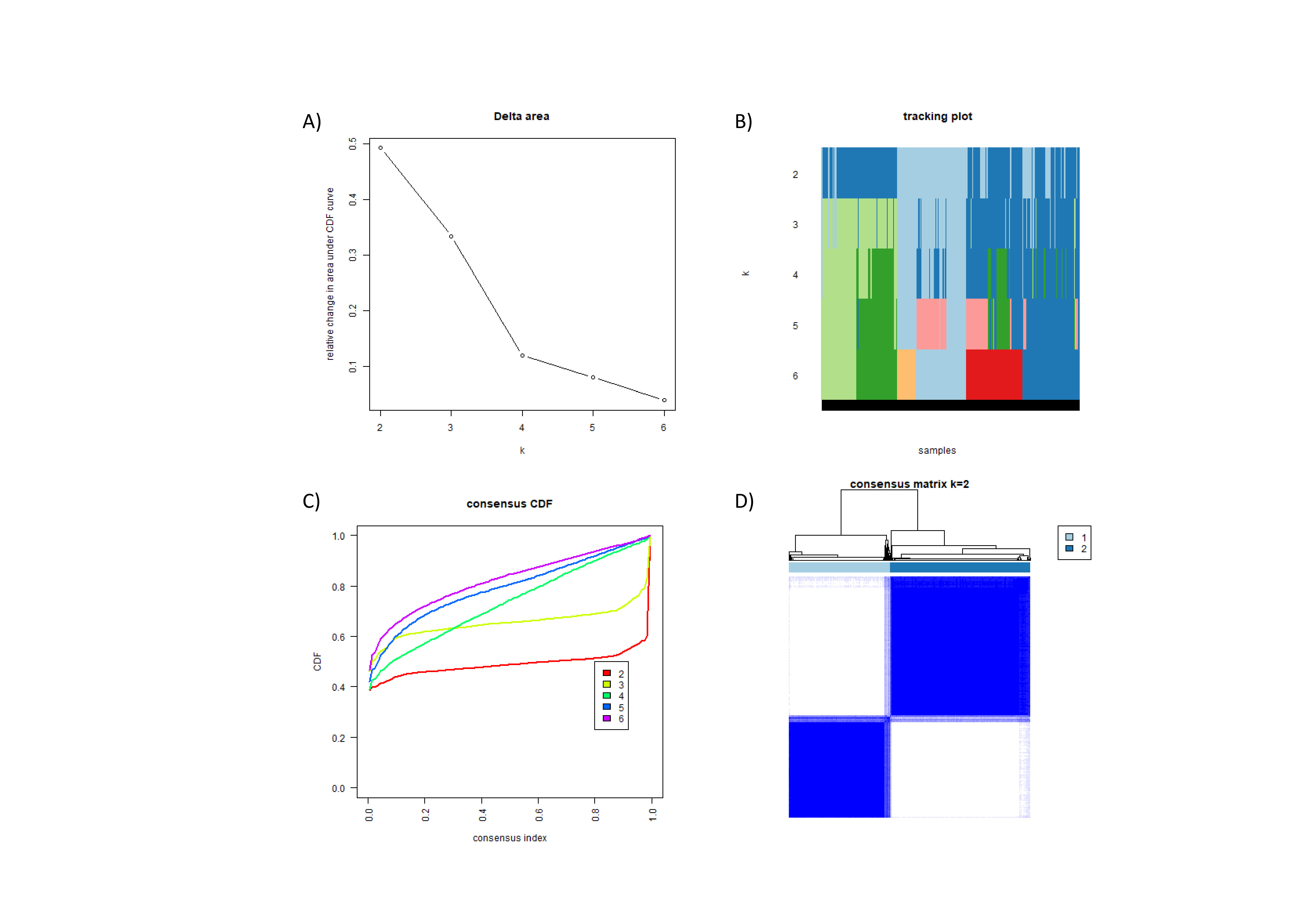

Supplement: Supplementary file 5 — Additional file 5: Fig. S2. Consensus clustering using k-means. A) Relative change in the area under the cumulative distribution function (CDF) curve with increasing clusters (k), with little change beyond k = 2, B) Tracking plot across k = 2–6, C) Consensus CDF plot across. [file 13054_2024_4837_MOESM5_ESM.jpg]
